# Mammographically Occult Breast Cancer in a Patient With Dense Breast Tissue

**DOI:** 10.7759/cureus.77789

**Published:** 2025-01-21

**Authors:** Arden J Bewersdorf, Emerson L Bewersdorf, Gina M Fundaro

**Affiliations:** 1 Biology, Detroit Country Day School, Beverly Hills, USA; 2 Biology, Northville High School, Northville, USA; 3 Radiology/Breast Imaging and Intervention, Regional Medical Imaging, Novi, USA

**Keywords:** breast cancer, dense breast tissue, mammography, mri, supplemental breast screening

## Abstract

Routine screening mammography can decrease the mortality from breast cancer by early detection. Dense breast tissue limits the detection of cancer with mammography alone, and the use of supplemental imaging to improve early detection is necessary in these patients. For women with dense breasts who would benefit from supplemental screening, modalities, such as MRI, whole breast ultrasound, contrast-enhanced mammography, and molecular breast imaging, are available to aid in the detection of mammographically occult cancers.

## Introduction

The purpose of screening for breast cancer is to detect cancer early to reduce mortality [1]. Data from the United States suggest that 47% of the screening population has dense breast tissues [2]. Breast tissue composition varies from predominantly fatty breast tissue to extremely dense breast tissue. Breast cancer risk has been found to be four to six times higher in women with extremely dense breast tissue compared to those with fatty breast tissue [2,3]. As breast tissue increases in density, mammographic sensitivity decreases. Mammographic sensitivity of 93% in fatty breasts decreases to as low as 30% in extremely dense breasts [3]. Breast cancer can be radiopaque on mammography and dense breast tissue is radiopaque on mammography, which contributes to the overall decrease in mammographic sensitivity as breast density increases. Dense breast tissue on screening mammography is associated with an increased incidence of breast cancer and higher mortality rates, which drives new considerations for screening in patients with dense breast tissue [3].

## Case presentation

**Figure 1:**
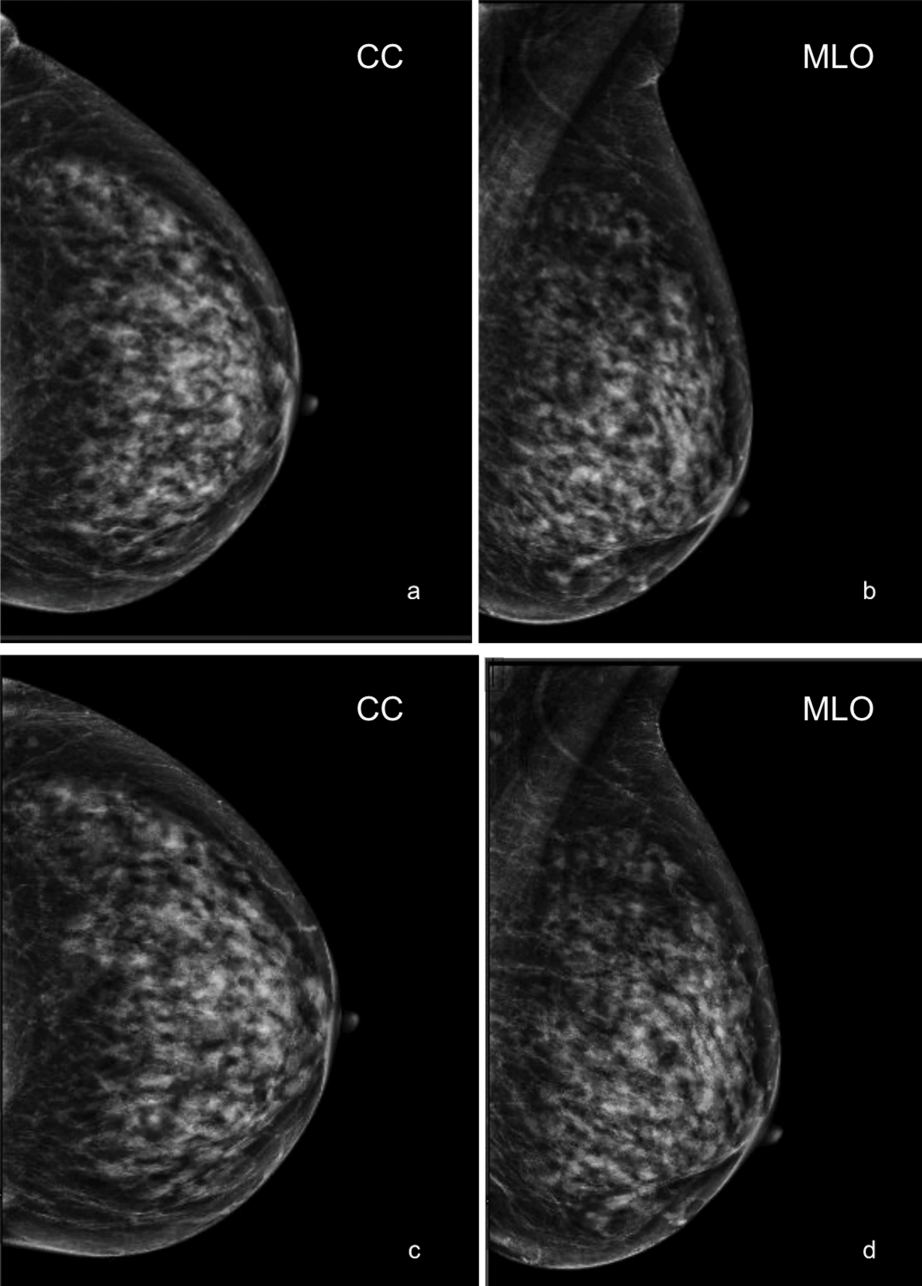
Negative mammograms with no significant changes. Craniocaudal (a) and mediolateral oblique (b) left breast screening mammograms performed in May 2023. Craniocaudal (c) and mediolateral oblique (d) left breast screening mammograms performed in May 2024. The breasts were extremely dense with no suspicious masses, areas of architectural distortion, or groups of microcalcifications. No significant changes were seen when comparing studies.

An 89-year-old asymptomatic female, with a sister and aunt having a history of breast cancer, presented for routine annual screening mammography in May 2024. A comparison of routine screening mammograms between May 2022 (not pictured) and May 2023 showed no significant changes (Figures 1a, 1b). Her breasts were extremely dense without evidence of a suspicious mass, area of architectural distortion, or group of microcalcifications (Figures 1c, 1d). There were no enlarged axillary lymph nodes.

**Figure 2:**
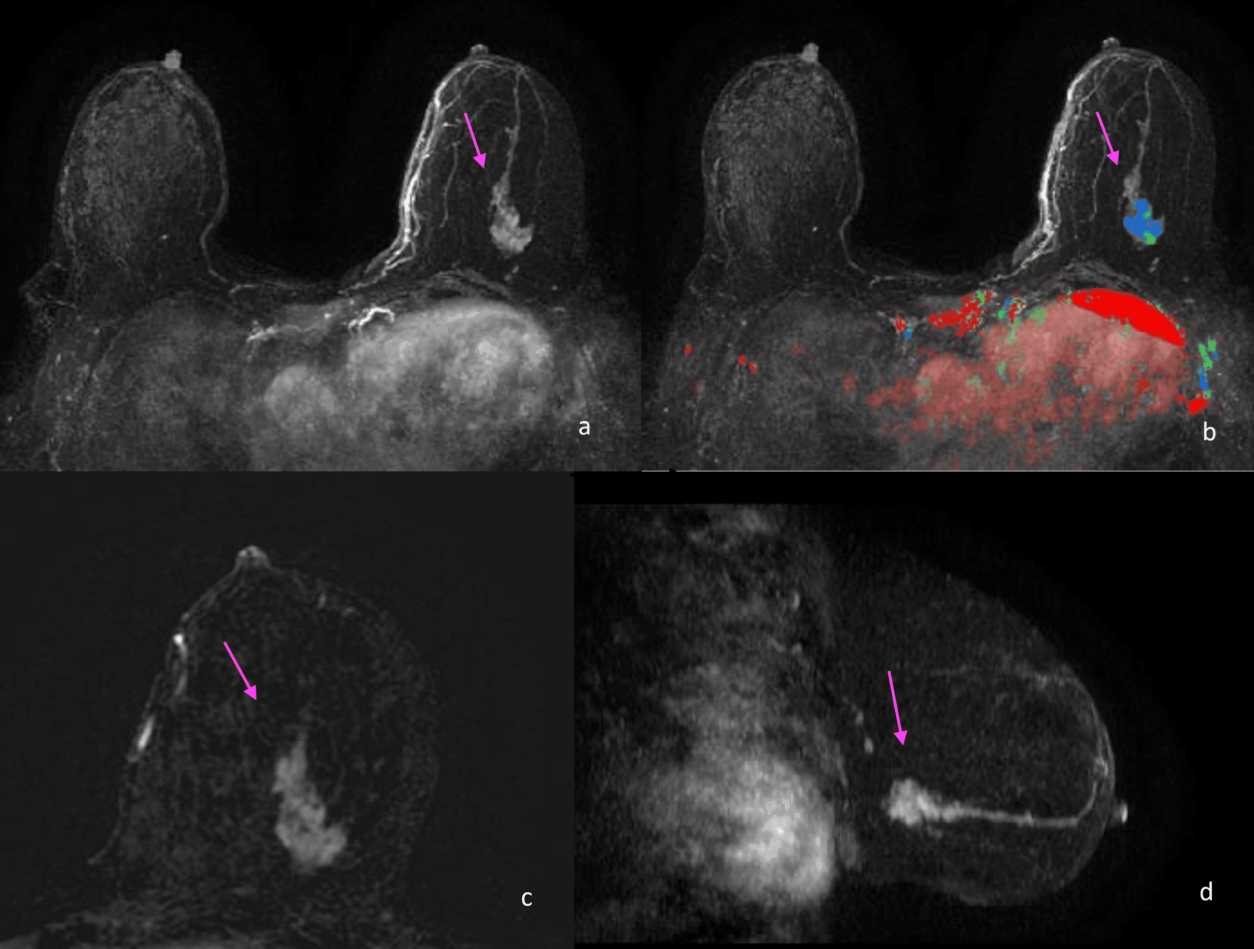
Contrast-enhanced MRI showing a 3.3 cm enhancing mass with irregular margins in the left breast. T1-weighted contrast-enhanced subtracted MRI axial (a-c) and sagittal (d) images obtained in November 2024 demonstrate a 3.3 cm enhancing mass with irregular margins in the inferior lateral left breast, extending from the middle to the posterior depth (arrows). Mixed kinetic enhancement was seen within the mass (b).

A supplemental screening study was recommended at the time of the negative mammogram in May 2024 due to extremely dense breast tissue and known family history. The patient underwent a contrast-enhanced screening breast MRI in November 2024. MRI demonstrates an enhancing mass of 3.3 cm with irregular margins in the inferior lateral left breast, extending from middle to posterior depth (Figures 2a-2d). Based on the MRI findings, an MRI-guided biopsy was performed.

**Figure 3:**
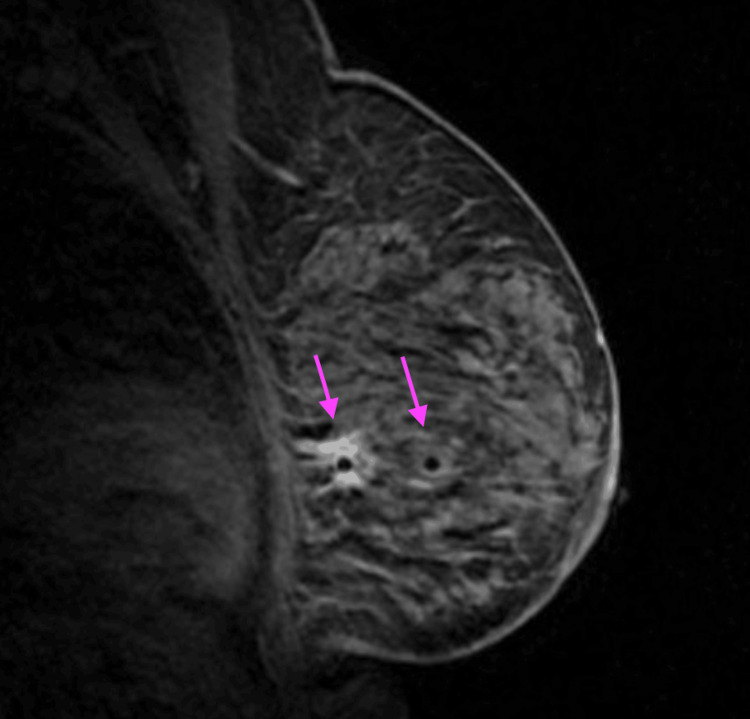
MRI-guided biopsy of the enhancing mass in the left breast. T1-weighted post-contrast, non-fat-saturated sagittal MRI intra-biopsy image demonstrated the placement of a biopsy needle in both the middle and posterior aspects of the mass (arrows).

**Figure 4:**
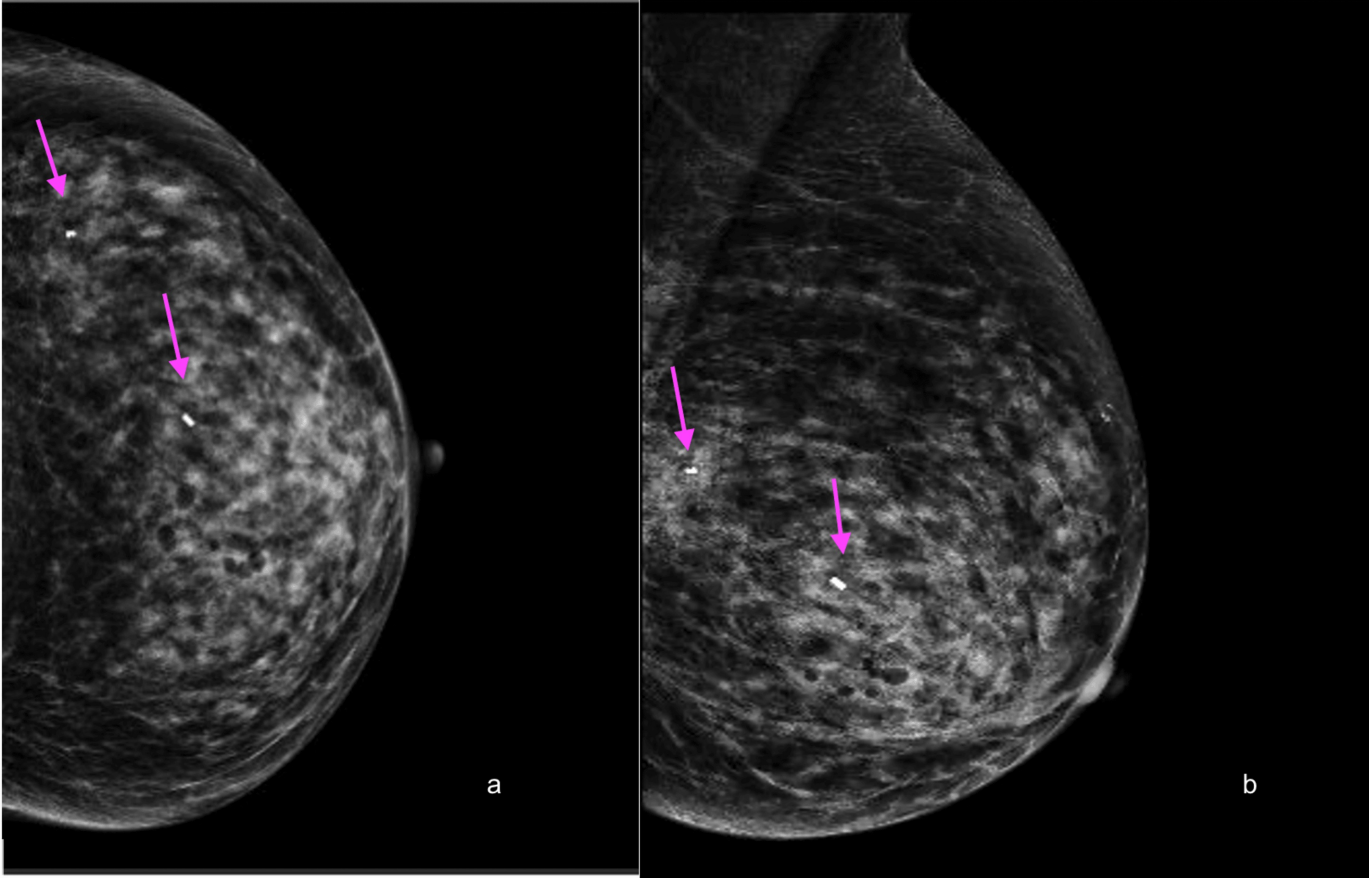
Post-procedure mammogram showing no suspicious findings at the biopsy site. Post-procedure craniocaudal (a) and mediolateral oblique (b) mammograms demonstrated the placement of metallic clip markers post-biopsy, located in the inferior lateral left breast at the middle and posterior depths (arrows).

MRI-guided biopsy was performed, sampling the middle and posterior aspects of the mass (Figure 3). Post-procedure metallic clips were seen in the inferior lateral left breast at the middle and posterior depth, marking both tissue sampling sites. There were no corresponding suspicious mammographic findings at the site of either MRI-guided biopsy clip when evaluating the post-procedure mammogram (Figures 4a, 4b). The pathologic diagnosis at both the middle and posterior biopsy site was invasive lobular carcinoma, intermediate grade, and estrogen receptor/progesterone receptor (ER/PR) positive.

## Discussion

Breast density ranging from least dense to most dense is described as fatty, scattered, and heterogeneous or extremely dense breast tissue [1]. Mammographic sensitivity decreases as breast density increases. Overall mammographic sensitivity is 79.9%, however, mammographic sensitivity of 93% in fatty breasts decreases to as low as 30% in extremely dense breasts [3]. Supplemental screening for patients with extremely dense breast tissue with imaging modalities in addition to mammography, such as whole breast ultrasound, contrast-enhanced mammography, and contrast-enhanced MRI may increase the detection of cancer [4].

The use of supplemental MRI screening in extremely dense breast patients who had a negative mammogram resulted in the diagnosis of significantly fewer interval cancers than mammography alone during a two-year screening period according to the DENSE clinical trial [5]. Multiple prospective studies performed on women at higher than average risk demonstrate that breast MRI has a higher sensitivity than mammography, ultrasound, and combined screening with mammography and ultrasound [6]. For most women at higher-than-average risk, the supplemental screening modality of choice is contrast-enhanced MRI [6]. Although MRI is more sensitive as a supplemental screening modality, consideration must be given that not all patients will have equal access to MRI including high body mass patients, claustrophobic patients, patients with difficulty lying prone, and rural and low-income patients [3]. In such situations where MRI is of limited availability or contraindicated, supplemental screening with ultrasound, contrast-enhanced mammography, or molecular breast imaging can be considered. Evidence supports the ability of supplemental screening modalities, such as MRI, whole breast ultrasound, contrast-enhanced mammography, and molecular breast imaging, to detect incremental cancers after negative mammographic screening in dense breast patients [7].

## Conclusions

Routine screening mammography can decrease the mortality from breast cancer by early detection. However, the sensitivity of mammography alone can be as low as 30% in patients with dense breast tissues. Supplemental screening modalities have been shown to detect more breast cancer than mammography alone in patients with dense breast tissue and should be used in addition to mammography to increase the sensitivity of detecting breast cancer in these patients.
